# Natural variation in cross-talk between glucosinolates and onset of flowering in *Arabidopsis*

**DOI:** 10.3389/fpls.2015.00697

**Published:** 2015-09-08

**Authors:** Lea M. Jensen, Henriette S. K. Jepsen, Barbara A. Halkier, Daniel J. Kliebenstein, Meike Burow

**Affiliations:** ^1^Department of Plant and Environmental Sciences, Faculty of Science, DNRF Center DynaMo, University of CopenhagenFrederiksberg, Denmark; ^2^Department of Plant and Environmental Sciences, Faculty of Science, Copenhagen Plant Science Centre, University of CopenhagenFrederiksberg, Denmark; ^3^Department of Plant Sciences, University of California, DavisDavis, CA, USA

**Keywords:** glucosinolates, AOP2, AOP3, flowering time, regulation, cross-talk

## Abstract

Naturally variable regulatory networks control different biological processes including reproduction and defense. This variation within regulatory networks enables plants to optimize defense and reproduction in different environments. In this study we investigate the ability of two enzyme-encoding genes in the glucosinolate pathway, *AOP2* and *AOP3*, to affect glucosinolate accumulation and flowering time. We have introduced the two highly similar enzymes into two different *AOP*^*null*^ accessions, Col-0 and Cph-0, and found that the genes differ in their ability to affect glucosinolate levels and flowering time across the accessions. This indicated that the different glucosinolates produced by AOP2 and AOP3 serve specific regulatory roles in controlling these phenotypes. While the changes in glucosinolate levels were similar in both accessions, the effect on flowering time was dependent on the genetic background pointing to natural variation in cross-talk between defense chemistry and onset of flowering. This variation likely reflects an adaptation to survival in different environments.

## Introduction

To maximize plant fitness in challenging, ever-changing and unpredictable environments, organisms must coordinate growth and defense to respond to diverse combinations of biotic and abiotic factors. Interactions between biotic and abiotic factors force growth and defense to co-evolve for local adaptation. Optimizing this necessary co-evolution requires the combination of various mechanistic solutions including the plasticity provided by regulatory networks to respond to environmental changes. Additional solutions are provided by the diversity inherent in genetic variation within a plant species, consequently providing different solutions depending on the environment (Pigliucci, 2003). Together, regulatory networks and genetic variation establish the potential for diverse solutions across a species to optimize growth and defense across highly varied environments (Burow et al., 2010; Paul-Victor et al., 2010; Woods et al., 2012).

**Figure 1:**
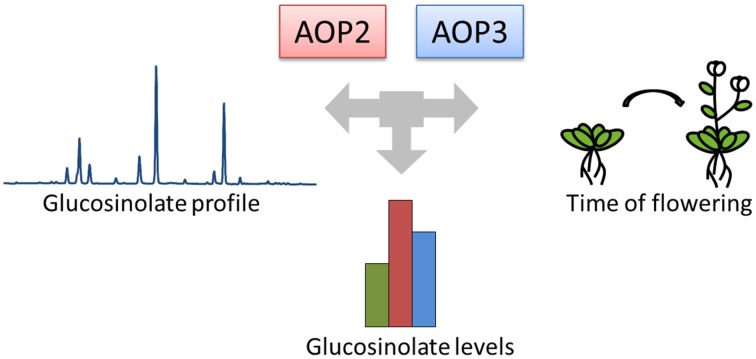
***AOP2* and *AOP3* have been associated with natural variation in different phenotypes**. The *GS-AOP* locus encoding the glucosinolate biosynthetic enzymes AOP2 and AOP3 has been associated with variation in glucosinolate profiles due to their enzymatic activities. The same genes have been linked to changes in glucosinolate levels and onset of flowering in different natural variation studies.

To identify genes involved in cross-talk between growth and defense, we focused on *Arabidopsis* natural variation studies. Numerous studies have identified key genes controlling natural variation in the plastic timing of flowering time and thereby reproduction (Koornneef et al., 1991; Michaels and Amasino, 1999; Johanson et al., 2000; El-Din El-Assal et al., 2001; Salomé et al., 2011; Ward et al., 2012; Grillo et al., 2013; Méndez-Vigo et al., 2013). Similarly, natural variation studies in the primary *Arabidopsis* defense compounds, the glucosinolates produced from tryptophan (indole glucosinolates) and methionine (aliphatic glucosinolates), have aimed at understanding the diversity in glucosinolate profiles (Kliebenstein et al., 2001a,b; Hirai et al., 2005; Keurentjes et al., 2006; Wentzell et al., 2007; Rowe et al., 2008; Jensen et al., 2014). Cross-talk between the networks controlling flowering and glucosinolate profiles seems to occur, as glucosinolate biosynthetic genes within the *GS-AOP* locus have been associated with not only glucosinolate biosynthesis, but also the control of onset of flowering (Figure 1), (Atwell et al., 2010; Kerwin et al., 2011). Hence, the genes in this locus may help illuminate the cross-talk between defense and flowering time in *Arabidopsis*.

**Figure 2:**
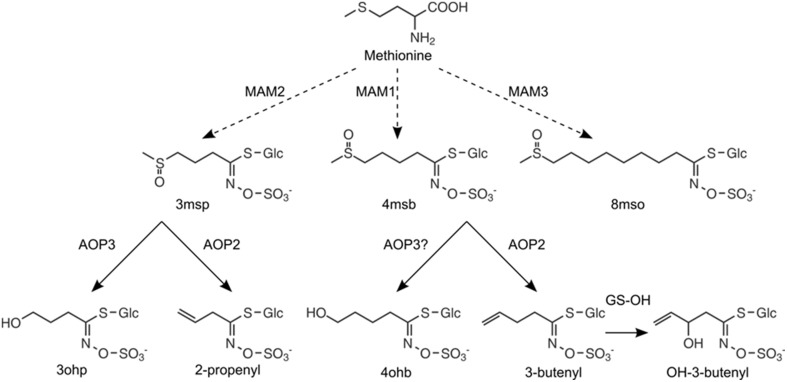
**Enzymatic functions of MAMs and AOPs in the aliphatic glucosinolate pathway**. The chain length of aliphatic glucosinolates is controlled by *GS-ELONG:* expression of *MAM2* in the absence of *MAM1* leads to C3 glucosinolates, *MAM1* is required for the production of C4 glucosinolates, and *MAM3* is responsible for the production of LC glucosinolates with C8 as the predominant chain length. The C3 glucosinolate, 3-methylsulfinylpropyl glucosinolate (3msp), can be converted to 3-hydroxypropyl glucosinolate (3ohp) by AOP3 or to 2-propenyl glucosinolate (2-prop) by AOP2. The C4 glucosinolate, 4-methylsulfinylbutyl glucosinolate (4msb), is converted to 3-butenyl by AOP2 glucosinolate (3-but), which is further converted by GS-OH to 2(*R*/*S*)-hydroxy-3-butenyl glucosinolate. Here, we report that 4msb can also be converted by AOP3 to give 4-hydrozybutyl glucosinolate (4ohb), (see **Figure 3**).

The *GS-AOP* locus encodes the two 2-oxoglutarate-dependent dioxygenases AOP2 and AOP3, which convert specific short-chained (SC) methylsulfinylalkyl glucosinolate precursors to alkenyl glucosinolates and hydroxyalkyl glucosinolates, respectively (Figure 2), (Mithen et al., 1995; Kliebenstein et al., 2001c). While AOP2 catalyzes the conversion of the 3-methylsulfinylpropyl (3msp) to the 2-propenyl glucosinolate as well as the conversion of 4-methylsulfinylbutyl (4msb) to 3-butenyl glucosinolate, AOP3 activity has only been detected for 3msp, which is converted by AOP3 to form 3-hydroxypropyl glucosinolate (3ohp), (Kliebenstein et al., 2001c). Natural variation in sequence and expression patterns of *AOP2* and *AOP3* leads to different glucosinolate profiles among *Arabidopsis* accessions. Studies investigating natural *AOP* alleles have linked the expression of an enzymatically functional *AOP2* or *AOP3* in leaves to increased glucosinolate content, however, with different regulatory potential (Kliebenstein et al., 2001b; Wentzell et al., 2007; Rohr et al., 2009, 2012). Overall, accessions expressing a functional AOP2 enzyme show the highest glucosinolate levels, followed by *AOP3* accessions, which still accumulate glucosinolates to higher levels than *AOP*^*null*^ accessions (Kliebenstein et al., 2001b).

Based on the catalytic properties of AOP2 and AOP3, we hypothesized that the previously observed difference in these two genes' potential regulatory effects may depend on their enzymatic function and substrate availability, i.e., the levels of 3msp and 4msb. The production of these substrates is largely controlled by the allelic status of *GS-ELONG* encoding different methylthioalkylmalate synthases. In the absence of a functional *AOP2* or *AOP3*, the presence of *MAM1* (methylthioalkylmalate synthase 1) leads to accumulation of 4msb as the major SC aliphatic glucosinolate, while accumulation of 3msp is attributed to the presence of *MAM2* (Figure 2), (de Quiros et al., 2000; Kroymann et al., 2001, 2003). *GS-ELONG* and *GS-AOP* show an epistatic interaction for glucosinolate accumulation (Kliebenstein et al., 2001b). This epistasis might be linked to the differences in AOP2/3 substrate availability depending on the allelic state at *GS-ELONG*, which would hint at specific feedback effects of the different products formed by AOP2 and AOP3.

In addition to their enzymatic and regulatory role in glucosinolate biosynthesis, both *AOP2* and *AOP3* have also been linked to flowering time control. Introduction of a functional AOP2 to the *AOP*^*null*^ accession Col-0 shortened the circadian clock period and altered flowering time (Kerwin et al., 2011). In contrast, the *AOP3* gene was found to have a hit in a genome wide association study to the control of the transcript level for a key flowering regulator, the MADS-box transcription factor *FLC* (Flowering Locus C), (Atwell et al., 2010). Yet the associations between the *AOPs* and flowering time remain to be tested in different accessions. However, *AOP2* and *AOP3* may mediate cross-talk between chemical defense i.e., glucosinolates and onset of flowering, which is critical for adaptation to environmental settings.

Hence, we tested if these two enzyme encoding genes, *AOP2* and *AOP3*, have different effects on glucosinolate levels and flowering time in two different accessions, Col-0 and Cph-0, which express neither AOP2 nor AOP3. We introduced *AOP2* and *AOP3* into the two *AOP*^*null*^ accessions that differ at the *GS-ELONG* locus and thus accumulate different AOP substrates. The use of multiple transgenic lines enable direct comparison of the effects of the three different *AOP* alleles (*AOP2, AOP3, AOP*^*null*^) on glucosinolate accumulation and flowering time. Thus, this approach allowed us to systematically investigate the differences in the regulatory roles of all three *AOP* alleles, which is not possible in natural variation studies such as GWAS or in QTL mapping e.g., using RILs or F2 populations. The introduction of *AOP2* and *AOP3* led to different changes in the glucosinolate profiles in the two accessions. While the regulatory effects on glucosinolate levels were similar in both accessions, our study shows that *AOP2* and *AOP3* possess different abilities to change flowering time dependent on the genetic background.

## Materials and methods

### Generation of expression constructs

For generation of expression constructs, we extracted genomic DNA from leaf tissue using the CTAB method (Clarke, 2009). The genomic sequence of *AOP2* was amplified from Col-0 gDNA expressing the *AOP2* allele of *B. oleracea BoGSL-ALK* under the control of the CaMV 35S promoter (Li and Quiros, 2003) with the primers 5′-ggcttaauATGGGTG CAGACACTCCTCAAC-3′ and 5′-ggtttaauTTATGCT CCAGAGACGGCAC-3′. *AOP3* was amplified from L*er* gDNA using primers designed based on the reference sequence from TAIR9, 5′-ggcttaauATGGGTTCATGCAGTCCTCA-3′ and 5′-ggtttaauTTATTTCCCAGCAGAGACGC-3′. *AOP2* and *AOP3* were inserted into pCAMBIA2300-35Su and pCAMBIA3300-35Su, respectively, downstream of the CaMV 35S promoter (Nour-Eldin et al., 2006). The constructs were verified by sequencing.

### Generation of transgenic plants

*Agrobacterium tumefaciens* (strain PGV38 c58) was transformed with either the 35S:AOP2 or the 35S:AOP3 expression construct for transformation of Col-0 and Cph-0 by floral-dip (Clough and Bent, 1998). Positive transformants carrying the 35S:AOP2 construct were selected on MS plates with 50 μM kanamycin. T1 seeds carrying the 35S:AOP3 construct were selected by repeated spraying with 300 μM Basta at the 4-leaf stage. In the T1 and subsequent generations, the presence of the transgenes was confirmed by PCR on genomic DNA. For *AOP2*, we obtained multiple insertion lines in both Col-0 and Cph-0, whereas for *AOP3* multiple lines were obtained in Cph-0, but only one line in Col-0.

### Plant growth

For all experiments, seeds were sown in a randomized design and cold stratified at 4°C for at least 2 days. The plants were grown in climate chambers at 80–120 μE/(m^2*^ s) light intensity, 16 h light, 20°C, and 70% relative humidity. Flowering time was measured as the period between cold stratification and the day, when the inflorescence reached 1 cm in height and normalized to the corresponding WT.

### Glucosinolate analysis

Glucosinolates were extracted from fresh mature rosette leaves around 22 (Col-0) or 27 (Cph-0) days after stratification and analyzed as desulfo-glucosinolates for some experiments by HPLC/DAD as previously described (Kliebenstein et al., 2001c; Andersen et al., 2013) using p-hydroxybenzyl glucosinolate as an internal standard. Separation of desulfo-glucosinolates by HPLC was achieved on a Supelcosil LC-18-DB column, 25 cm × 4.6 mm, 5 μm particle size (Supelco, Bellefonte, PA, USA) or a ZORBAX SB-Aq column, 25 cm × 4.6 mm, 5 μm particle size (Agilent Technologies).

Alternatively, desulfo-glucosinolates were analyzed by UHPLC/TQ-MS on an Advance™-UHPLC/EVOQ™Elite-TQ-MS instrument (Bruker) equipped with a C-18 reversed phase column (Kinetex 1.7 u XB-C18, 10 cm × 2.1 mm, 1.7 μm particle size, Phenomenex) by using a 0.05% formic acid in water (v/v) (solvent A)-0.05% formic acid in acetonitrile (v/v) (solvent B) gradient at a flow rate of 0.4 ml/min at 40°C. The gradient applied was as follows: 2% B (0.5 min), 2–30% (0.7 min), 30–100% (0.8 min), 100% B (0.5 min), 100–102% B (0.1 min), and 2% B (1.4 min). Compounds were ionized by ESI with a spray voltage of +3500 V, heated probe temperature 400°C, cone temperature 250°C.

Desulfo-glucosinolates were monitored based on the following MRM transitions: 3-methylthiopropyl (3mtp), (+)328 > 166 [5V]; 3-methylsulfinyl (3msp), (+)344 > 182 [10V]; 2-propenyl (2-prop), (+)280 > 118 [5V]; 3-hydroxypropyl (3ohp), (+)298 > 118 [15V]; 4-methylthiobutyl (4mtb), (+)342 > 132 [15V]; 4-methylsulfinylbutyl (4msb), (+)358 > 196 [5V]; 3-butenyl (3-but), (+)294 > 132 [15V]; 2*R*/2*S*-2-hydroxy-3-butenyl, (+)310 > 130 [15V]; 4-hydroxybutyl (4ohb), (+)312 > 132 [15V]; 5-methylsulfinylpentyl (5msp), (+)372 > 210 [5V]; 7-methylthioheptyl (7mth), (+)384 > 222 [5V]; 7-methylsulfinylheptyl (7msh), (+)400 > 238 [7V]; 8-methylthiooctyl (8mto), (+)398 > 236 [5V]; 8-methylsulfinyloctyl (8mso), (+)414 > 252 [5V]; indol-3-ylmethyl (I3M), (+)369 > 207 [10V]; *N*-methoxy-indol-3-ylmethyl (NMOI3M), (+)399>237 [10V]; 4-methoxy-indol-3-ylmethyl (4MOI3M), (+)399 > 237 [10V]; and p-hydroxybenzyl (pOHB), (+)346 > 184 [10V] (internal standard). 2*R*- and 2*S*-2-hydroxy-3-butenyl glucosinolate and *N*- and 4-methoxy-indol-3-ylmethyl glucosinolate were distinguished based on retention times in comparison to those of known standards. Quantification of the individual glucosinolates was based on response factors relative to pOHB calculated from standard curves in control extracts.

### Statistics

We used R version 3.0.1 (2013-05-16) for statistical analysis (Team, 2013). For the WT and insertion lines significance was tested using the lm and Anova function for the following linear model GLS = Experiment + Genotype + insertion line nested within Genotype + Experiment:Genotype with specific differences tested *post-hoc* using the pairwise *t*-test function with a Holm-adjustment for multiple testing. Summary statistics was calculated using the SummaryBy from the doBy package (Højsgaard and Halekoh, 2014).

### Generation of the Col-0 × Cph-0 F2 population

To create the Col-0 × Cph-0 F2 mapping population, Col-0 and Cph-0 WT plants were grown until the flowering stage and Cph-0 was used to pollinate Col-0. The F1 plants were genotyped for *MAM1* and *MAM2* to insure the presence of a copy of each allele, i.e., heterozygosity, before the F1 plants were selfed. The F2 population was investigated for flowering time by measuring the number of days from stratification till the primary inflorescence reached 1 cm (File S1 in Supplementary Table 1). For 171 plants, we obtained both genotype and phenotype results, which could be used in QTL mapping.

### Genotyping by MassARRAY

For genotyping, genomic DNA was extracted using the Qiagen DNeasy 96 Plant Kit (Qiagen) according to manufacturer's protocol. 100 sites were chosen for Sequenom MassARRAY in an attempt to get full coverage of the genome. However, we did not have any previous knowledge on the Cph-0 accession and only 53 of the SNPs turned out being polymorphic between the parents. These SNPs were used to generate genetic maps for each mapping population using the Haldane function (File S2 in Supplementary Table 1).

### QTL mapping

Windows QTL Cartographer Version 2.5 was used for composite interval mapping determining significant thresholds for flowering time by doing 1000 permutations to estimate the 0.05 significance levels (Wang et al., 2012). The main-effect markers were validated and tested for Two-Way epistatic interactions using lm type II and ANOVA in R version 3.0.1 (2013-05-16) including the most significant marker for each QTL.

### Quantitative real-time PCR

RNA was extracted from three pools of leaves for each genotype with Sigma Spectrum Plant Total RNA kit, treated with Sigma DNAse1 and reverse transcribed with iScript (Bio-rad). Expression was assayed by quantitative real-time PCR using SYBR Green and the data was normalized to UBC expression of each pool. The following primers were used: *UBC* (At5g25760), 5′-CTGAGCCGGACA GTCCTCTTAACTG-3′ and 5′-CGGCGAGGC GTGTATACATTTGTG 3′; *FT*, 5′-TGGTGGAGA AGACCTCAGGA-3′ and 5′-GAGGTGAGGGTT GCTAGGAC-3′; *FLC* in Col-0, 5′-GAGCCAAGAA GACCGAACTCAT-3′ and 5′-GAGATTTGTCCA GCAGGTGAC-3′; *FLC* in Cph-0: 5′-TGACTAGAG CCAAGAAGACCG-3′ and 5′-AGCAGGTGA CATCTCCATCTC-3′. Gene expression levels are presented as mean fold difference for two individual experiments. Results obtained in two independent experiments were pooled for statistical analysis lm. Anova and pairwise *t*-test with holm adjustment was used to test for significant differences at each time point and between experiments in R version 3.0.1 (2013-05-16) (Team, 2013).

## Results

### *AOP2* and *AOP3* alter glucosinolate profiles in Col-0 and Cph-0

Biosynthesis of the glucosinolate core structure gives rise to methylthioalkyl glucosinolates, which are further converted to methylsulfinylalkyl glucosinolates by GS-OX FMO1-5 (Hansen et al., 2007; Sønderby et al., 2010). Methylsulfinylalkyl glucosinolates accumulate in *AOP*^*null*^ accessions, i.e., accessions that express neither a functional *AOP2* nor *AOP3* in leaves (Figure 2). To investigate the relative effects of *AOP2* and *AOP3*, we introduced them to two *AOP*^*null*^ accessions accumulating different SC methylsulfinylalkyl glucosinolates. We used the Col-0 accession accumulating mainly 4msb and a so far undescribed accession accumulating 3msp as its major SC glucosinolate, Copenhagen-0 (Cph-0).

**Figure 3:**
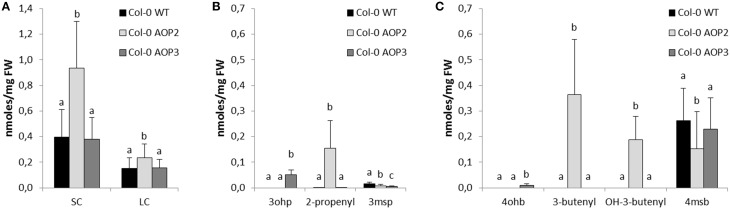
**Effects of *AOP2* and *AOP3* on glucosinolate accumulation in Col-0**. Glucosinolate concentrations in leaves of Col-0 WT (black), *n* = 28, Col-0 *AOP2* (light gray), *n* = 18 (2 independent insertion lines), and Col-0 *AOP3* (dark gray), *n* = 25 (1 line), as **(A)** Total SC and LC, **(B)** C3 glucosinolates, and **(C)** C4 substrate and products. Means (+ standard deviations) are shown for two experimental repeats of the same lines. Nested ANOVA across independent lines and post testing was used for statistical analysis. Letters indicate significant differences (*P* < 0.05) between genotypes.

In agreement with previous work using the *Brassica oleracea* or *Arabidopsis* accession Pi *AOP2* (Li and Quiros, 2003; Wentzell et al., 2007; Neal et al., 2010), constitutive expression of *AOP2* in the Col-0 background lead to the formation of 2-propenyl and 3-butenyl glucosinolate from 3msp to 4msb, respectively (Figures 2, 3B,C, for all individual glucosinolates see Figure S1). The presence of a functional GS-OH within Col-0 further modified the 3-butenyl side chain to 2*R*- and 2*S*-2-hydroxy-3-butenyl (Figure 3C), (Hansen et al., 2008). *AOP3* expression in Col-0 led to the conversion of 3msp to 3ohp and interestingly, we also detected small amounts of 4-hydroxybutyl glucosinolate (4ohb), (Figures 3B,C). Thus, AOP3 appears to be able to convert 4msb to 4ohb *in planta*, an activity that could not be detected *in vitro* (Kliebenstein et al., 2001c). However, based on the ratios of substrates and products in Col-0, AOP3 seems to have a preference for 3msp.

**Figure 4:**
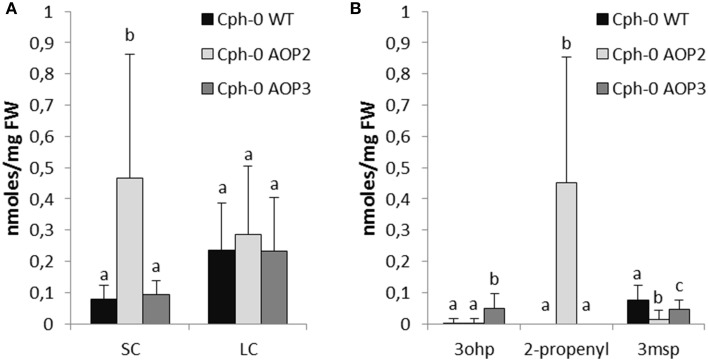
**Effects of *AOP2* and *AOP3* on glucosinolate accumulation in Cph-0**. Glucosinolate concentrations in leaves of Cph-0 WT including empty vector controls (black), *n* = 104, Cph-0 AOP2 (light gray), *n* = 73 (3 independent insertion lines), and Cph-0 AOP3 (dark gray), *n* = 60 (3 independent insertion lines), as **(A)** Total SC and LC, and **(B)** The C3 substrate and products. Means (+ standard deviations) are shown for analysis of two experimental repeats of the lines. Nested ANOVA across independent lines and post testing was used for statistical analysis. Letters indicate significant differences (*P* < 0.05) between genotypes.

In the Cph-0 background, 4msb is absent and instead, 3msp is the major SC glucosinolate. Consequently, introduction of the enzymes into the Cph-0 led to fewer different glucosinolate structures than in Col-0. As expected, *AOP2* expression resulted in the formation of 2-propenyl glucosinolate from 3msp, whereas AOP3 formed 3ohp from the same substrate (Figure 4B, for all individual glucosinolates see Figure S1).

### *AOP2* and *AOP3* have differential effects on glucosinolate levels in Col-0 and Cph-0

Several studies have suggested that both *AOP2* and *AOP3* influence the total level of glucosinolate accumulation as well as the specific structures being produced (Kliebenstein et al., 2001b; Wentzell et al., 2007; Rohr et al., 2009, 2012; Brachi et al., 2015). To directly compare the regulatory capacity of both genes, we quantified the glucosinolates in our different *AOP2* and *AOP3* lines. The introduction of *AOP2* to Col-0 led to a several fold increase in SC glucosinolates (Figure 3A), which is in agreement with previously published studies (Wentzell et al., 2007; Burow et al., 2015). The high levels of SC glucosinolates correlate with high accumulation of glucosinolates down-stream of AOP2, i.e., 2-propenyl, 3-butenyl, and 2*R*/2*S*-2-hydroxy-3-butenyl (Figures 3B,C). Introduction of *AOP2* to Col-0 does, however, not only change the levels of SC but also LC glucosinolates, which are not AOP2 substrates (Figure 3A). In contrast to *AOP2*, the introduction of *AOP3* into Col-0 did not change the total accumulation of SC or LC glucosinolates (Figure 3A). Thus, in the Col-0 background, *AOP2* and *AOP3* might differ in their ability to regulate glucosinolate accumulation possibly depending on their difference in enzymatic properties. However, as only one *AOP3* line was obtained in Col-0, we cannot completely rule out a regulatory effect of *AOP3* on glucosinolate accumulation in this background. In this line, *AOP3* expression caused a larger decrease in 3msp than *AOP2* and 4msb levels were only significantly decreased in the *AOP2* lines (Figures 3B,C). This difference may be critical in case the removal of substrates is important for the regulatory role through a feed-back mechanism. However, if the regulatory role of *AOP2* depends on its enzymatic activity, not only the removal of substrates but also the accumulation of the specific glucosinolate products may mediate this function.

Next, we investigated potential regulatory effects of *AOP2* and *AOP3* in Cph-0 that accumulates 3msp as the major SC glucosinolate. Cph-0 expressing *AOP2* accumulated high levels of SC glucosinolates (Figure 4A). Similar to the effect of *AOP2* expression in Col-0, increased SC glucosinolate levels correlated with the amounts of the AOP2 product (Figure 4B). In Cph-0, AOP2 only converted 3msp to 2-propenyl, suggesting that the effect of *AOP2* on SC may not depend on the chain length of the available substrate. In contrast to Col-0, introduction of *AOP2* to Cph-0 did not significantly affect LC glucosinolate accumulation. Thus, the regulatory effect of *AOP2* on LC glucosinolates might differ between the two accessions. Similar to the Col-0 accession, *AOP3* did not change the levels of total SC or LC glucosinolates in Cph-0 (Figure 4A). Even though the AOP2 and AOP3 can both convert 3msp, AOP2 seems to more efficiently pull from the 3msp pool than AOP3 and possibly thereby increase the flux through the pathway in the Cph-0 accession. Taken together, *AOP2* has a larger effect on the regulation of glucosinolate levels than *AOP3* in both accessions, which might be explained by the difference in their catalytic activities.

### Effects on flowering time differ between *AOP2* and *AOP3* and depend on the genetic background

**Figure 5:**
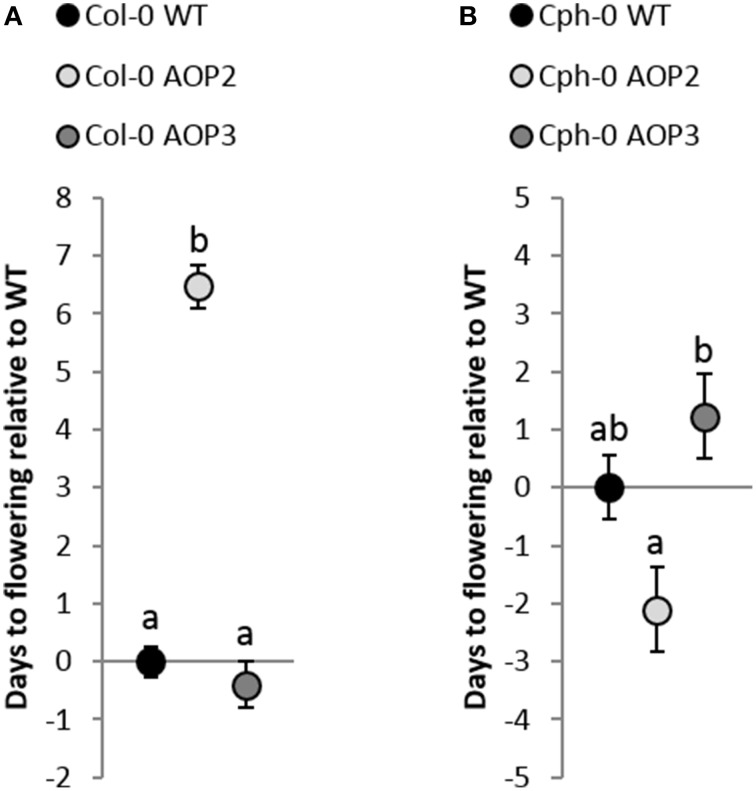
**Effects of *AOP2* and *AOP3* on flowering**. **(A)** Average (+ standard error) of flowering time in days relative to Col-0 WT (28.4 days ± 5.3). Black Col-0 WT, *n* = 110, light gray Col-0 *AOP2*, *n* = 50, (2 independent insertion lines), and dark gray Col-0 *AOP3*, *n* = 32, (1 line). ANOVA with nesting and experiment interaction (min 2 repeats) shows that Col-0 *AOP2* is significantly different from Col-0 WT and Col-0 *AOP3*, *P* < 0.001, whereas *P* = 0.45 for the Col-0 WT and Col-0 *AOP3* comparison. **(B)** Flowering time relative to Cph-0 WT (41.6 days ±5.3). Cph-0 WT (black), *n* = 60, Cph-0 *AOP2* (light gray), *n* = 73 (3 independent insertion lines), and Cph-0 *AOP3* (dark gray), *n* = 60 (3 independent insertion lines). ANOVA with nesting of the different insertion lines and experiment interaction (two repeats) showed no significant difference between Cph-0 WT and the insertion lines; Cph-0 WT and Cph-0 *AOP2* (*P* = 0.06) and Cph-0 WT and Cph-0 *AOP3* (*P* = 0.22). Cph-0 *AOP2* and Cph-0 *AOP3* showed a significant difference (*P* < 0.01).

The glucosinolate biosynthetic genes *AOP2* and *AOP3* represent candidate genes for the integration of defense with reproduction as they are associated with the control of flowering time in both the laboratory and the field (Atwell et al., 2010; Kerwin et al., 2011, 2015). To test the ability of *AOP2* and *AOP3* to link glucosinolates and flowering time in different backgrounds, we measured flowering time in all of our lines (Figure 5). *AOP2* has been identified as a QTL for altering circadian clock parameters and thereby flowering time (Kerwin et al., 2011). Accordingly, introduction of a functional *AOP2* into Col-0 under 16 h light delayed flowering time by several days (Figure 5A). *AOP3* has been associated with natural variation in flowering time and gene expression level of the MADS-box transcription factor *FLC* (Flowering Locus C), which is one of the major determinants of flowering (Shindo et al., 2005; Atwell et al., 2010). Analysis of the Col-0 *AOP3* line showed no significant difference between Col-0 WT and Col-0 *AOP3* lines (Figure 5A). Thus, *AOP2* but not *AOP3* seems to influence onset of flowering in Col-0.

In contrast to its pronounced delay on flowering in Col-0, *AOP2* had a suggestive ability to speed up flowering in Cph-0 using three independent transgenic lines (nested ANOVA; *P* = 0.06; Figure 5B). Similarly, *AOP3* expression in the Cph-0 background did not significantly change flowering time compared to the WT (Figure 5B). However, the *AOP2* and *AOP3* lines in Cph-0 showed a significant difference in flowering time from each other (*P* < 0.01), indicating that *AOP2* and *AOP3* have significant opposite effects on flowering time in Cph-0, which differs from the effects in Col-0.

### Flowering time network architecture is critical for the regulatory effects of *AOP2* and *AOP3*

While *AOP2* and *AOP3* had similar effects on glucosinolate accumulation across Col-0 and Cph-0, each gene differs in its ability to alter flowering time in the two accessions. One factor that may contribute to this difference is variation in the internal flowering time pathways in the two accessions. The Col-0 WT flowers earlier than Cph-0 WT indicating differences in the flowering networks. To identify the underlying loci that contribute to these differences and the variation in the *AOP2/3* effect on flowering, we established a Col-0 × Cph-0 F2 population to map QTLs that control the major difference in flowering time between Col-0 and Cph-0. We genotyped 171 F2 plants for 100 SNPs using Sequenom MassARRAY® and this resulted in identification of 53 polymorphic sites. For the Cph-0 accession with no prior sequence knowledge, we compared the SNP combination to the 1001 genome accession database (Cao et al., 2011; Schneeberger et al., 2011; Long et al., 2013) and did not find any annotated accession to have the same combination. The F2 population was also phenotyped for flowering time.

**Figure 6:**
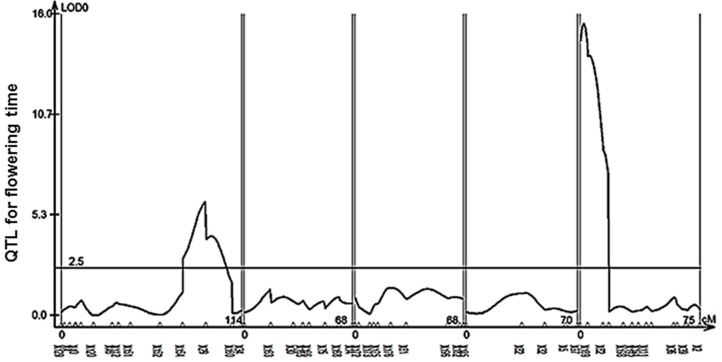
**QTL mapping for flowering time in the Col-0 × Cph-0**. QTL mapping in an F2 population derived from a Col-0 × Cph-0 cross revealed two significant loci (X129 and X188) controlling flowering time.

QTL mapping for flowering time revealed two loci (X29 and X188) as the major QTLs (Figure 6, Table 1). We found *FT* and *FLC* as the top candidate genes in these loci (candidate gene list from Grillo et al., 2013). Therefore, we analyzed the transcript levels of *FT* and *FLC* in the two accessions and found *FT* expression in Cph-0 to be 1–2% of the transcript level in Col-0, while *FLC* transcript levels were around 500 times higher in Cph-0 than in Col-0 (Table 2). This is in agreement with the observed difference in flowering time between the two accessions, as *FLC* delays flowering time by repressing *FT* expression. This difference suggests that the ability to detect the influence of the *AOP2* and *AOP3* genes on flowering may depend on the allelic status at these known major effect flowering time genes in *Arabidopsis*. Further work is needed to assess how the *AOP2* or *AOP3* genes interplay with these known flowering time genes.

**Table 1:** **ANOVA table for flowering time QTLs**.

**Markers**	**Sum Sq**	**Df**	***F*-Value**	***P*-Value**
X29	4011.8	2	9.4698	1.4 E-4
X188	14891.3	2	35.1503	5.4 E-13
X29: X188	686.4	4	0.8101	0.52

**Table 2:** **Expression analysis for flowering time genes in QTLs identified in the Col-0 × Cph-0 F2 population**.

**AGI**	**Gene**	**Time**	**Fold difference**	***P*-Value**
AT1G65480	FT	Day 22	0.012	1.2 E-5
		Day 28	0.026	1.9 E-4
AT5G10140	FLC	Day 22	581	3.0 E-5
		Day 28	484	4.3 E-5

Fold difference indicates expression levels in Cph-0 relative to Col-0.

## Discussion

In the field, glucosinolate profiles and herbivory resistance are strongly dependent on *GS-AOP* and *GS-ELONG* (Bidart-Bouzat and Kliebenstein, 2008; Züst et al., 2012; Brachi et al., 2015; Kerwin et al., 2015). Hence, the structures and levels of the glucosinolates produced depend on the allelic status of these two loci and their interaction plays an important role for plant fitness. The introduction of *AOP2* into the *AOP*^*null*^ accessions Col-0 and Cph-0 causes accumulation of alkenyl glucosinolates together with increased levels of aliphatic glucosinolates (Figures 3, 4), (Li and Quiros, 2003; Wentzell et al., 2007; Burow et al., 2015). In contrast, *AOP3* expression in the same backgrounds led to formation of hydroxyalkyl glucosinolates without associated changes in glucosinolate levels. Thus, the production of alkenyl but not hydroxyalkyl glucosinolates influences the feedback regulation of glucosinolate biosynthesis in Cph-0 and possibly also in Col-0. Both AOP2 and AOP3 convert the same substrates, which suggests that it is the product being produced and not the substrate that is the determining factor. Consequently, the increased flux from primary metabolism into specialized metabolism in the presence of *AOP2* may depend on the products of the enzyme mediating positive feedback regulation of the pathway. Recent work has suggested that this may occur by altering the jasmonate signal transduction pathway in lines that contain a functional *AOP2* gene (Burow et al., 2015).

*AOP2* significantly increased levels of aliphatic glucosinolates in both Col-0 and Cph-0 that differ in their *GS-ELONG* allelic status. This suggests that variation in the presence of 3C or 4C substrate availability is not the major determinant for the control of SC glucosinolates by the interaction of *AOP2* and *GS-ELONG*. Only in the Col-0 background, LC glucosinolate levels were also significantly increased in the *AOP2* lines, which could suggest that this effect requires the presence of C4 glucosinolates or variation in the *MAM3*, which catalyzes the production of the LC glucosinolates. The fact that LC levels differ between the two WTs suggests that there is *MAM3* variation or in the regulatory network controlling LC glucosinolate accumulation.

As previously found, we could show that *AOP2* delays flowering time in Col-0 (Kerwin et al., 2011), but there was only a suggestive effect of *AOP2* on flowering in the Cph-0 background (Figure 5). Mapping flowering time variation between Col-0 and Cph-0 suggested that these two accessions have natural variation in *FLC* and *FT* suggesting that this difference in the *AOP2* effect on flowering time may be due to interactions with the known flowering time pathways. Supporting this hypothesis, *AOP2* has been shown to alter the circadian clock pathway that affects flowering time via regulation of *FT* (Kerwin et al., 2011). Thus, the regulatory effect of *AOP2* on flowering time in Col-0 through *FT* might be altered by the competing regulation by the 500 times higher *FLC* levels in Cph-0. Recently, *AOP2* was moreover found to mediate positive feedback regulation between the aliphatic glucosinolate biosynthetic pathway and jasmonate signaling (Burow et al., 2015). More specifically, expression of *AOP2* in Col-0 led to increased transcript levels of *MYC2*. Interestingly, *MYC2* is not only a key regulator of jasmonate-mediated plant responses (Lorenzo et al., 2004; Dombrecht et al., 2007) including glucosinolate biosynthesis, but also involved in circadian oscillation of jasmonate signaling through direct interaction with the clock component TIME FOR COFFEE (Shin et al., 2012). The ability of *AOP2* to alter flowering time in Col-0 might thus be linked to its regulatory input into jasmonate signaling. In contrast to *AOP2*, no clear effect was observed for *AOP3* in either accession. Yet, *AOP3* has been linked to natural variation in *FLC* expression (Atwell et al., 2010) indicating that *AOP3* provides input to the flowering time network through a different mechanism than *AOP2*. If *AOP3* can play a regulatory role in flowering time at all, the effect is minor and possibly requires a specific allelic status at the flowering time genetic loci.

Our study illustrates natural variation in the cross-talk between glucosinolate accumulation and flowering time. The ability to fine-tune this cross-talk differs between the two enzyme-encoding genes, *AOP2* and *AOP3*, even though they arose from a recent gene duplication event and share high sequence similarity (Kliebenstein et al., 2001c). To fully understand the role of the *GS-AOP* locus in fine-tuning the regulatory cross-talk between glucosinolate profiles, jasmonate signaling, and the onset of flowering time, future studies in a larger number of genetic backgrounds will be required. Most importantly, studies in accessions expressing a functional *AOP2* or *AOP3* will reveal potential differences in the architecture of the regulatory networks in these backgrounds. Nevertheless, the specific regulatory effects and the dependency on the genetic background possibly reflect the plant's need to coordinate defense and reproduction when faced with different combinations of biotic and abiotic challenges. Hence, *Arabidopsis* seems to have evolved different cross-talk mechanisms linking defense and flowering time phenotypes to adapt to different environments.

## Author contributions

LJ, DK, and MB designed the study and interpreted the data. LJ and MB conducted the plant work. LJ, MB, and DK did the statistical analyses. HJ designed and carried out expression analyses. LJ and MB wrote the paper. DK and BH commented on the manuscript.

### Conflict of interest statement

The authors declare that the research was conducted in the absence of any commercial or financial relationships that could be construed as a potential conflict of interest.

## References

[B1] Andersen T. G. Nour-Eldin H. H. Fuller V. L. Olsen C. E. Burow M. Halkier B. A. (2013). Integration of biosynthesis and long-distance transport establish organ-specific glucosinolate profiles in vegetative arabidopsis. Plant Cell 25, 3133–3145. 10.1105/tpc.113.110890 23995084 PMC3784604

[B2] Atwell S. Huang Y. S. Vilhjalmsson B. J. Willems G. Horton M. Li Y.. (2010). Genome-wide association study of 107 phenotypes in *Arabidopsis thaliana* inbred lines. Nature 465, 627–631. 10.1038/nature08800 20336072 PMC3023908

[B3] Bidart-Bouzat M. G. Kliebenstein D. J. (2008). Differential levels of insect herbivory in the field associated with genotypic variation in glucosinolates in *Arabidopsis thaliana*. J. Chem. Ecol. 34, 1026–1037. 10.1007/s10886-008-9498-z 18581178

[B4] Brachi B. Meyer C. G. Villoutreix R. Platt A. Morton T. C. Roux F.. (2015). Coselected genes determine adaptive variation in herbivore resistance throughout the native range of *Arabidopsis thaliana*. Proc. Natl. Acad. Sci. U.S.A. 112, 4032–4037. 10.1073/pnas.1421416112 25775585 PMC4386350

[B5] Burow M. Atwell S. Francisco M. Kerwin R. E. Halkier B. A. Kliebenstein D. J. (2015). The glucosinolate biosynthetic gene Aop2 mediates feed-back regulation of jasmonic acid signaling in *Arabidopsis*. Mol. Plant 8, 1201–1212. 10.1016/j.molp.2015.03.001 25758208

[B6] Burow M. Halkier B. A. Kliebenstein D. J. (2010). Regulatory networks of glucosinolates shape *Arabidopsis thaliana* fitness. Curr. Opin. Plant Biol. 13, 348–353. 10.1016/j.pbi.2010.02.002 20226722

[B7] Cao J. Schneeberger K. Ossowski S. Gunther T. Bender S. Fitz J.. (2011). Whole-genome sequencing of multiple *Arabidopsis thaliana* populations. Nat. Genet. 43, 956–963. 10.1038/ng.911 21874002

[B8] Clarke J. D. (2009). Cetyltrimethyl ammonium bromide (CTAB) DNA miniprep for plant DNA isolation. Cold Spring Harb Protoc. 3:Pdb.prot5177. 10.1101/pdb.prot5177 20147112

[B9] Clough S. J. Bent A. F. (1998). Floral dip: a simplified method for *Agrobacterium*-mediated transformation of *Arabidopsis thaliana*. Plant J. 16, 735–743. 10.1046/j.1365-313x.1998.00343.x 10069079

[B10] de Quiros H. C. Magrath R. McCallum D. Kroymann J. Scnabelrauch D. Mitchell-Olds T.. (2000). Alpha-keto acid elongation and glucosinolate biosynthesis in *Arabidopsis thaliana*. Theor. Appl. Genet. 101, 429–437. 10.1007/s00122005150025851613

[B11] Dombrecht B. Xue G. P. Sprague S. J. Kirkegaard J. A. Ross J. J. Reid J. B.. (2007). MYC2 differentially modulates diverse jasmonatedependent functions in *Arabidopsis*. Plant Cell 19, 2225–2245. 10.1105/tpc.106.048017 17616737 PMC1955694

[B12] El-Din El-Assal S. Alonso-Blanco C. Peeters A. J. Raz V. Koornneef M. (2001). A QTL for flowering time in *Arabidopsis* reveals a novel allele of CRY2. Nat. Genet. 29, 435–440. 10.1038/ng767 11726930

[B13] Grillo M. A. Li C. Hammond M. Wang L. Schemske D. W. (2013). Genetic architecture of flowering time differentiation between locally adapted populations of *Arabidopsis thaliana*. New Phytol. 197, 1321–1331. 10.1111/nph.12109 23311994

[B14] Hansen B. G. Kerwin R. E. Ober J. A. Lambrix V. M. Mitchell-Olds T. Gershenzon J.. (2008). A novel 2-oxoacid-dependent dioxygenase involved in the formation of the goiterogenic 2-hydroxybut-3-enyl glucosinolate and generalist insect resistance in *Arabidopsis*. Plant Physiol. 148, 2096–2108. 10.1104/pp.108.129981 18945935 PMC2593654

[B15] Hansen B. G. Kliebenstein D. J. Halkier B. A. (2007). Identification of a flavin-monooxygenase as the S-oxygenating enzyme in aliphatic glucosinolate biosynthesis in *Arabidopsis*. Plant J. 50, 902–910. 10.1111/j.1365-313X.2007.03101.x 17461789

[B16] Hirai M. Y. Klein M. Fujikawa Y. Yano M. Goodenowe D. B. Yamazaki Y.. (2005). Elucidation of gene-to-gene and metabolite-to-gene networks in *Arabidopsis* by integration of metabolomics and transcriptomics. J. Biol. Chem. 280, 25590–25595. 10.1074/jbc.M502332200 15866872

[B17] Højsgaard S. Halekoh U. (2014). DoBy: Groupwise Statistics, LSmeans, Linear Contrasts, Utilities. Aalborg.

[B18] Jensen L. M. Halkier B. A. Burow M. (2014). How to discover a metabolic pathway? An update on gene identification in aliphatic glucosinolate biosynthesis, regulation and transport. Biol. Chem. 395, 529–543. 10.1515/hsz-2013-0286 24589761

[B19] Johanson U. West J. Lister C. Michaels S. Amasino R. Dean C. (2000). Molecular analysis of FRIGIDA, a major determinant of natural variation in *Arabidopsis* flowering time. Science 290, 344–347. 10.1126/science.290.5490.344 11030654

[B20] Kerwin R. E. Jimenez-Gomez J. M. Fulop D. Harmer S. L. Maloof J. N. Kliebenstein D. J. (2011). Network quantitative trait loci mapping of circadian clock outputs identifies metabolic pathway-to-clock linkages in *Arabidopsis*. Plant Cell 23, 471–485. 10.1105/tpc.110.082065 21343415 PMC3077772

[B21] Kerwin R. Feusier J. Corwin J. Rubin M. Lin C. Muok A.. (2015). Natural genetic variation in *Arabidopsis thaliana* defense metabolism genes modulates field fitness. Elife 4:e05604. 10.7554/eLife.05604 25867014 PMC4396512

[B22] Keurentjes J. J. Fu J. de Vos C. H. Lommen A. Hall R. D. Bino R. J.. (2006). The genetics of plant metabolism. Nat. Genet. 38, 842–849. 10.1038/ng1815 16751770

[B23] Kliebenstein D. J. Gershenzon J. Mitchell-Olds T. (2001a). Comparative quantitative trait loci mapping of aliphatic, indolic and benzylic glucosinolate production in *Arabidopsis thaliana* leaves and seeds. Genetics 159, 359–370. Available online at: http://www.genetics.org/content/159/1/359.long 11560911 10.1093/genetics/159.1.359 PMC1461795

[B24] Kliebenstein D. J. Kroymann J. Brown P. Figuth A. Pedersen D. Gershenzon J.. (2001b). Genetic control of natural variation in *Arabidopsis* glucosinolate accumulation. Plant Physiol. 126, 811–825. 10.1104/pp.126.2.811 11402209 PMC111171

[B25] Kliebenstein D. J. Lambrix V. M. Reichelt M. Gershenzon J. Mitchell-Olds T. (2001c). Gene duplication in the diversification of secondary metabolism: tandem 2-oxoglutarate-dependent dioxygenases control glucosinolate biosynthesis in *Arabidopsis*. Plant Cell 13, 681–693. 10.1105/tpc.13.3.681 11251105 PMC135509

[B26] Koornneef M. Hanhart C. J. Van der veen J. H. (1991). A genetic and physiological analysis of late flowering mutants in *Arabidopsis thaliana*. Mol. Gen. Genet. 229, 57–66. 10.1007/BF00264213 1896021

[B27] Kroymann J. Donnerhacke S. Schnabelrauch D. Mitchell-Olds T. (2003). Evolutionary dynamics of an *Arabidopsis* insect resistance quantitative trait locus. Proc. Natl. Acad. Sci. U.S.A. 100, 14587–14592. 10.1073/pnas.1734046100 14506289 PMC304123

[B28] Kroymann J. Textor S. Tokuhisa J. G. Falk K. L. Bartram S. Gershenzon J.. (2001). A gene controlling variation in arabidopsis glucosinolate composition is part of the methionine chain elongation pathway. Plant Physiol. 127, 1077–1088. 10.1104/pp.010416 11706188 PMC129277

[B29] Li G. Quiros C. F. (2003). In planta side-chain glucosinolate modification in *Arabidopsis* by introduction of dioxygenase *Brassica* homolog BoGSL-ALK. Theor. Appl. Genet. 106, 1116–1121. 10.1007/s00122-002-1161-4 12671761

[B30] Long Q. Rabanal F. A. Meng D. Huber C. D. Farlow A. Platzer A.. (2013). Massive genomic variation and strong selection in *Arabidopsis thaliana* lines from Sweden. Nat. Genet. 45, 884–890. 10.1038/ng.2678 23793030 PMC3755268

[B31] Lorenzo O. Chico J. M. Sánchez-Serrano J. J. Solano R. (2004). JASMONATE-INSENSITIVE1 encodes a MYC transcription factor essential to discriminate between different jasmonate-regulated defense responses in *Arabidopsis*. Plant Cell 16, 1938–1950. 10.1105/tpc.022319 15208388 PMC514172

[B32] Méndez-Vigo B. Martínez-Zapater J. M. Alonso-Blanco C. (2013). The flowering repressor SVP underlies a novel *Arabidopsis thaliana* QTL interacting with the genetic background. PLoS Genet. 9:e1003289. 10.1371/journal.pgen.1003289 23382706 PMC3561112

[B33] Michaels S. D. Amasino R. M. (1999). FLOWERING LOCUS C encodes a novel MADS domain protein that acts as a repressor of flowering. Plant Cell 11, 949–956. 10.1105/tpc.11.5.949 10330478 PMC144226

[B34] Mithen R. Clarke J. Lister C. Dean C. (1995). Genetics of aliphatic glucosinolates.III. Side-chain structure of aliphatic glucosinolates in *Arabidopsis-thaliana*. Heredity 74, 210–215. 10.1038/hdy.1995.2922336876

[B35] Neal C. S. Fredericks D. P. Griffiths C. A. Neale A. D. (2010). The characterisation of AOP2: a gene associated with the biosynthesis of aliphatic glucosinolates in *Arabidopsis thaliana*. BMC Plant Biol. 10:170. 10.1186/1471-2229-10-170 20699011 PMC3095303

[B36] Nour-Eldin H. H. Hansen B. G. Nørholm M. H. H. Jensen J. K. Halkier B. A. (2006). Advancing uracil-excision based cloning towards an ideal technique for cloning PCR fragments. Nucleic Acids Res. 34, e122. 10.1093/nar/gkl635 17000637 PMC1635280

[B37] Paul-Victor C. Züst T. Rees M. Kliebenstein D. J. Turnbull L. A. (2010). A new method for measuring relative growth rate can uncover the costs of defensive compounds in *Arabidopsis thaliana*. New Phytol. 187, 1102–1111. 10.1111/j.1469-8137.2010.03325.x 20561205

[B38] Pigliucci M. (2003). Selection in a model system: ecological genetics of flowering time in *Arabidopsis thaliana*. Ecology 84, 1700–1712. 10.1890/0012-9658(2003)084[1700:SIAMSE]2.0.CO;2

[B39] Rohr F. Ulrichs C. Mewis I. (2009). Variability of aliphatic glucosinolates in *Arabidopsis thaliana* (L.) - impact on glucosinolate profile and insect resistance. J. Appl. Bot. Food Quality 82, 131–135. Available online at: http://pub.jki.bund.de/index.php/JABFQ/article/view/2091/2476

[B40] Rohr F. Ulrichs C. Schreiner M. Zrenner R. Mewis I. (2012). Responses of *Arabidopsis thaliana* plant lines differing in hydroxylation of aliphatic glucosinolate side chains to feeding of a generalist and specialist caterpillar. Plant Physiol. Biochem. 55, 52–59. 10.1016/j.plaphy.2012.03.005 22543106

[B41] Rowe H. C. Hansen B. G. Halkier B. A. Kliebenstein D. J. (2008). Biochemical networks and epistasis shape the *Arabidopsis thaliana* metabolome. Plant Cell 20, 1199–1216. 10.1105/tpc.108.058131 18515501 PMC2438456

[B42] Salomé P. A. Bomblies K. Laitinen R. A. E. Yant L. Mott R. Weigel D. (2011). Genetic architecture of flowering-time variation in *Arabidopsis thaliana*. Genetics 188, 421–433. 10.1534/genetics.111.126607 21406681 PMC3122318

[B43] Schneeberger K. Ossowski S. Ott F. Klein J. D. Wang X. Lanz C.. (2011). Reference-guided assembly of four diverse *Arabidopsis thaliana* genomes. Proc. Natl. Acad. Sci. U.S.A. 108, 10249–10254. 10.1073/pnas.1107739108 21646520 PMC3121819

[B44] Shin J. Heidrich K. Sanchez-Villarreal A. Parker J. E. Davis S. J. (2012). TIME FOR COFFEE represses accumulation of the MYC2 transcription factor to provide time-of-day regulation of jasmonate signaling in *Arabidopsis*. Plant Cell 24, 2470–2482, 10.1105/tpc.111.095430 22693280 PMC3406923

[B45] Shindo C. Aranzana M. J. Lister C. Baxter C. Nicholls C. Nordborg M.. (2005). Role of FRIGIDA and FLOWERING LOCUS C in determining variation in flowering time of *Arabidopsis*. Plant Physiol. 138, 1163–1173. 10.1104/pp.105.061309 15908596 PMC1150429

[B46] Sønderby I. E. Geu-Flores F. Halkier B. A. (2010). Biosynthesis of glucosinolates - gene discovery and beyond. Trends Plant Sci. 15, 283–290. 10.1016/j.tplants.2010.02.005 20303821

[B47] Team R. C. (2013). R: A Language and Environment for Statistical Computing. Vienna: R Foundation for Statistical Computing.

[B48] Wang S. Basten C. Zeng Z. (2012). Windows QTL Cartographer 2.5. Raleigh, NC: Department of Statistics, North Carolina State University.

[B49] Ward J. K. Samanta Roy D. Chatterjee I. Bone C. R. Springer C. J. Kelly J. K. (2012). Identification of a major QTL that alters flowering time at elevated [CO(2)] in *Arabidopsis thaliana*. PLoS ONE 7:e49028. 10.1371/journal.pone.0049028 23185291 PMC3504057

[B50] Wentzell A. M. Rowe H. C. Hansen B. G. Ticconi C. Halkier B. A. Kliebenstein D. J. (2007). Linking metabolic QTLs with network and cis-eQTLs controlling biosynthetic pathways. PLoS Genet. 3, 1687–1701. 10.1371/journal.pgen.0030162 17941713 PMC1976331

[B51] Woods E. C. Hastings A. P. Turley N. E. Heard S. B. Agrawal A. A. (2012). Adaptive geographical clines in the growth and defense of a native plant. Ecol. Monogr. 82, 149–168. 10.1890/11-1446.1

[B52] Züst T. Heichinger C. Grossniklaus U. Harrington R. Kliebenstein D. J. Turnbull L. A. (2012). Natural enemies drive geographic variation in plant defenses. Science 338, 116–119. 10.1126/science.1226397 23042895

