# Supplementary material for: Natural variation in cross-talk between glucosinolates and onset of flowering in Arabidopsis
Source: Front Plant Sci. 2015 Sep 8;6:697. doi: 10.3389/fpls.2015.00697 (PMC4561820; doi:10.3389/fpls.2015.00697)
Supplement: Supplementary file 2 [file Image1.PDF]

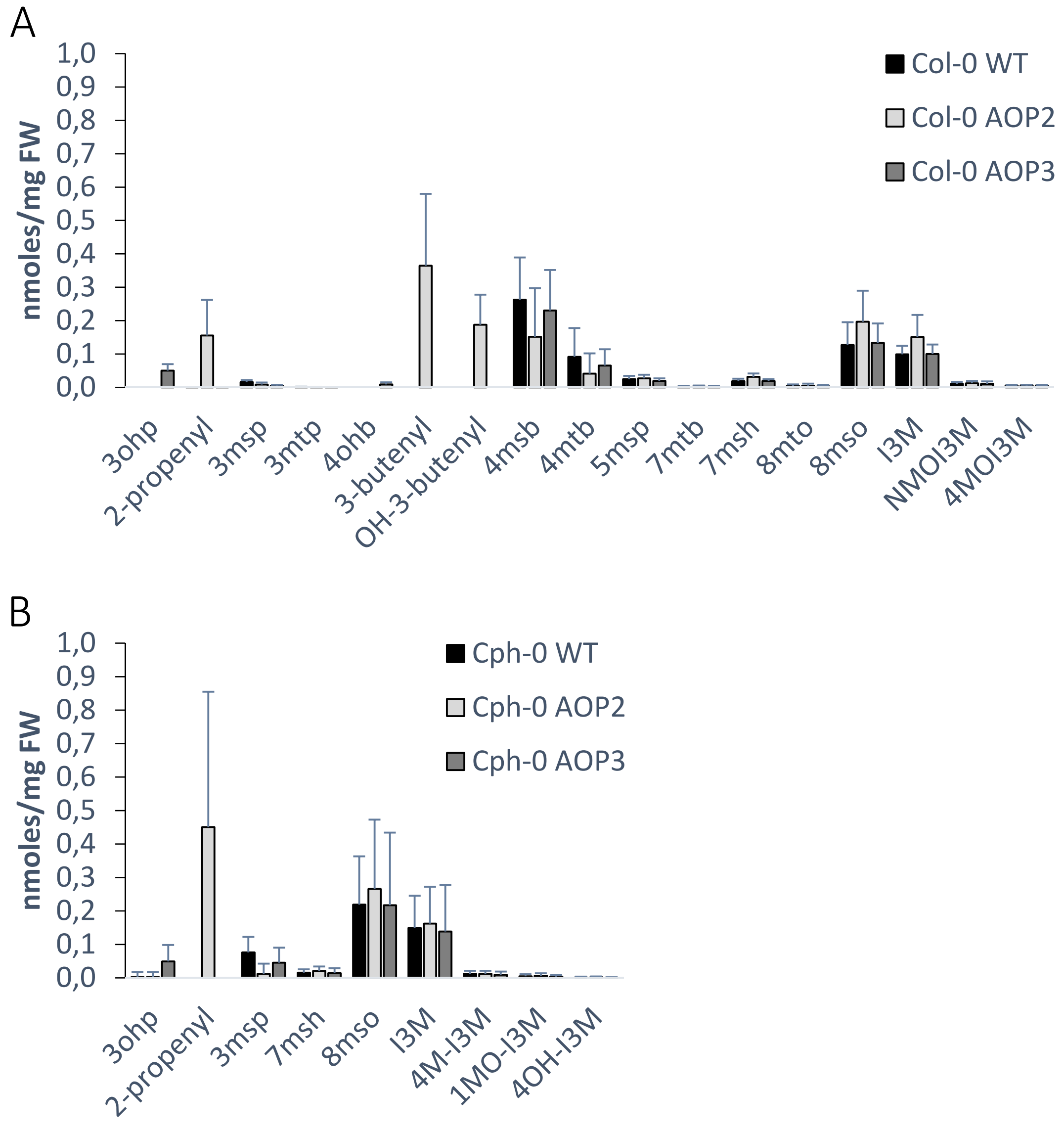

Figure S1 Individual glucosinolates in Col-0 and Cph-0 lines

Glucosinolates in leaves of Col-0 WT (black), n=28, Col-0 *AOP2* (light grey), n=18 (3 independent insertion lines), and Col-0 *AOP3* (dark grey), n=25 (1 line) **(A)**. Glucosinolate concentrations in leaves of Cph-0 WT including empty vector controls (black), n=104, Cph-0 AOP2 (light grey), n=73 (2 independent insertion lines), and Cph-0 AOP3 (dark grey), n=60 (3 independent insertion lines) **(B)**. Means (+ standard deviations) are shown for analysis of two experimental repeats of the lines
